# Awareness of the dual-use dilemma in scientific research: reflections and challenges to Latin America

**DOI:** 10.3389/fbioe.2025.1649781

**Published:** 2025-07-28

**Authors:** Jose Alonso Flores-Coronado, Alondra Yamileth Alanis-Valdez, Maria Fernanda Herrera-Saldivar, Aldo Sebastian Flores-Flores, Jose Manuel Vazquez-Guillen, Reyes S. Tamez-Guerra, Cristina Rodriguez-Padilla

**Affiliations:** Universidad Autónoma de Nuevo León, Facultad de Ciencias Biológicas, Laboratorio de Inmunología y Virología, San Nicolás de los Garza, Nuevo León, Mexico

**Keywords:** biosafety, biosecurity, dual-use research, ethics research, risk assessment

## Abstract

The dual-use dilemma compels us to reflect on scientific responsibility, ethical regulation, and the role of society in the governance of knowledge. While science has the potential to transform the world for the better, it can also become a double-edged sword if the implications of its potential misuse are overlooked. This is particularly relevant in the life sciences, where advances can be repurposed for harmful by secondary actors. Addressing this challenge requires the development of preventive tools, biosafety and biosecurity measures specifically designed for dual-use research, and clear, up-to-date regulatory frameworks at both national and international levels. These mechanisms should aim to anticipate risks, restrict unauthorized access to sensitive information, and foster a culture of responsibility within the scientific community, without hindering the advancement of knowledge or obstructing legitimate innovation. This perspective explores the dual-use dilemma through a global lens, with a specific focus on Latin America, a region where policies and institutional awareness on dual-use risks remain limited. By analyzing illustrative case studies, existing international tools, and regional gaps, this work highlights both the obstacles and opportunities for strengthening dual-use governance in Latin American research systems.

## Introduction

The dual-use dilemma arises when a single scientific effort can be applied for both beneficial and harmful purposes, and it is not always clear how to prevent misuse without compromising its positive potential. “Misuse” refers to any unethical or malicious application of the research, whether in civilian or military contexts (Miller and Selgelid, 2007). At its core, the dual-use dilemma is both an ethical and practical issue. It requires scientists not only to consider the ethical implications of their work, but also to evaluate whether alternative research approaches could achieve similar benefits while reducing potential risks. In this way, researchers must balance the pursuit of knowledge with the responsibility to avoid harm and the practicality of scientific decision-making. For researchers, their work may represent a meaningful breakthrough with no intention of causing damage. The dilemma, however, emerges when others—whether individuals, political or religious groups, or governments—repurpose that knowledge for harmful ends. This is the broader context in which the dual-use debate takes place.

In the life sciences, research has played a vital role in addressing health, social, economic, and environmental challenges, aiming to improve both quality of life and life expectancy. Yet the same knowledge can also be misapplied to harm humans, animals, or the environment (Selgelid, 2009). The dual-use dilemma involves two types of actors: original researchers, who conduct the work and apply it for its intended scientific purposes, and secondary users, who may reinterpret or repurpose the methods or results in unintended—and potentially harmful—ways. For decades, research in biology, chemistry, and physics has been recognized not only as a field of great benefit, but also as a potential source of significant risk if proper safeguards are not in place.

In Latin America, there is growing concern about the limited awareness, at institutional, educational, and governmental levels, regarding the importance of identifying and managing the dual-use potential of scientific research, particularly in the life sciences. The Global Health Security Index (GHSI), developed by the Nuclear Threat Initiative and the Johns Hopkins Center for Health Security, offers a useful snapshot of national health security capacities, including indicators on biosafety, biosecurity, and responsible science (Bell and Nuzzo, 2021). In its 2021 edition, the GHSI evaluated 195 countries across six categories and 37 indicators, using 171 questions. One of these indicators, “Dual-use research and culture of responsible science”, assesses whether a country promotes awareness and oversight of dual-use risks in scientific research. Most Latin American countries scored poorly on this indicator. For example, Brazil, one of the highest overall performers in the region, received a score of 33.3, while Mexico and Argentina received scores of 0.0. In contrast, the United States (US) and Canada scored 50.0, and the United Kingdom (UK) scored 33.3. While the GHSI provides valuable comparative data, its methodology has been criticized for overemphasizing publicly available documentation, favoring high-income countries (Razavi et al., 2020). Nonetheless, these findings suggest that many Latin American countries have yet to meaningfully integrate dual-use awareness into their national biosafety and biosecurity strategies, reflecting a broader regional gap in the governance of potentially sensitive scientific research.

Given this scenario, the present perspective aims to raise awareness of the dual-use dilemma in scientific research and to reflect on the challenges and opportunities for strengthening a culture of responsibility in Latin America. By analyzing illustrative case studies, international tools, and regional shortcomings, we seek to highlight the need for preventive mechanisms and institutional capacities to address dual-use risks, without hindering scientific progress.

## Reflective case examples on the dual-use dilemma

• Scientific Development and Weapons. The notion that scientific research can be used for both beneficial and harmful purposes is not new. Throughout history, both basic and applied research have contributed, sometimes inadvertently, to the development of weapons of mass destruction (WMDs). Some governments have supported scientific efforts to develop such weapons and, in some cases, have used them. For example, mustard gas, initially developed by Germany, was used during World War I and later reemerged during the Iraq-Iran conflict between 1980 and 1988. Similarly, the atomic bombs dropped by the US on Hiroshima and Nagasaki in 1945 caused between 150,000 and 246,000 deaths, most of them civilians. What began as a scientific exploration in nuclear physics ultimately led to one of the most devastating military application in history. Although nuclear weapons caused unprecedented destruction, other forms of scientific misuse continued. For instance, the Soviet Union maintained a large-scale biological weapons program from 1946 to 1992, illustrating how different scientific fields, including biology, can be exploited for harmful purposes.
• Genetic Sequencing and Pathogens. Sequencing the genomes or specific genes of pathogens has been essential for understanding their biology, enabling accurate diagnosis, and supporting the development of vaccines. However, open access to these sequences also opens the door to synthesizing pathogens artificially—potentially creating more virulent versions without needing natural samples (Sandbrink et al., 2023).
• The Mousepox Experiment. In 2001, a group of Australian researchers genetically modified the mousepox virus (Ectromelia virus; ECTV) to express interleukin-4 (IL-4), intending to induce infertility in mice as a pest control strategy. Unexpectedly, the modified virus turned out to be highly lethal—even in mice that had been previously vaccinated. The study, published in the Journal of Virology by the American Society for Virology (Jackson et al., 2001), has since become a widely-cited example of the dual-use dilemma, illustrating how legitimate biotechnological techniques can lead to unforeseen, hazardous outcomes. The article includes a detailed description of the materials and methods used, and one of the main concerns raised by this study was the possibility of using similar techniques to engineer more virulent forms of viruses that affect humans.
• Artificial Synthesis of Poliovirus. In 2002, researchers at Stony Brook University in New York, funded by the US Department of Defense, successfully synthesized poliovirus “from scratch”. Using the publicly available RNA genome and commercially sourced materials, they created a viable virus capable of paralyzing and killing mice. The study, published in Science (Cello et al., 2002), was intended to raise awareness about the risk of malicious actors manufacturing viruses without access to natural samples. One of the lead researchers, Eckard Wimmer, noted that while this synthesis took several years, advances in technology could enable the creation of far more complex pathogens much faster in the future.
• Use of Artificial Intelligence. The dual-use dilemma remains highly relevant in the age of artificial intelligence (AI). In 2023, a team of scientists using AI models for drug development unexpectedly generated multiple compounds with potential applications as chemical weapons. A passage from the published article states:

“*The thought had never struck us. We were vaguely aware of security concerns around work with pathogens or toxic chemicals, but that did not relate to us; we primarily operate in a virtual setting. Our work is rooted in building machine learning models for therapeutic and toxic targets to better assist in the design of new molecules for drug discovery. We have spent decades using computers and AI to improve human health—not to degrade it. We were naïve in thinking about the potential misuse of our trade, as our aim had always been to avoid molecular features that could interfere with the many different classes of proteins essential to human life. Even our projects on Ebola and neurotoxins, which could have sparked thoughts about the potential negative implications of our machine learning models, had not set our alarm bells ringing”* (Urbina et al., 2022).

Once they recognized the dual-use potential, the authors restricted access to the predictive model’s details and implemented risk mitigation measures. This case illustrates how AI tools, even when used *in silico*, may inadvertently lower the barrier to biological or chemical misuse.

As AI capabilities continue to advance rapidly in both technical power and accessibility, it is imperative that biosecurity frameworks evolve in parallel. This requires not only updating existing oversight mechanisms, but also the proactive development of governance strategies that are adaptive, scalable, and internationally harmonized. Such strategies must consider the dual-use potential of AI-enabled research, balancing scientific openness with risk mitigation.

• Dual Use in Universities. The dual-use dilemma is not exclusive to high-level research projects or those linked to public safety or human health. In 2018, a student team participating in the International Genetically Engineered Machines (iGEM) competition demonstrated that even educational projects can raise dual-use concerns. Although iGEM prohibits work with hazardous organisms, genetic parts, or activities that pose direct risks, this team’s project—focused on degrading electronic components to facilitate recycling—highlighted how their technology could potentially be used to destroy critical electronic equipment, such as computational systems. Acknowledging this risk, the team and their advisors took proactive measures to mitigate misuse and ensure the responsible project implementation (Millett et al., 2020).

To date, there are no well-documented public case studies of dual-use research of concern in Latin America, which may reflect the region’s limited awareness, insufficient reporting mechanisms, and weak institutional oversight. Nevertheless, the existence of research involving high-impact pathogens, advanced biotechnology, and synthetic biology, particularly in the fields of infectious disease and agriculture, suggests that dual-use risks are likely present. This lack of documented incidents should not be interpreted as an absence of vulnerability, but rather as a call to strengthen governance frameworks, incorporate dual-use risk assessments into research design, and promote a culture of scientific responsibility throughout the region.

As shown through the well-documented examples, the dual-use dilemma can emerge in diverse contexts, from experimental virology and synthetic biology to artificial intelligence and even student-led research. It is the scientific community’s responsibility to ensure that knowledge serves to protect—not endanger—human life, animal welfare, or the environment.

### International approaches to dual-use research of concern

Today, several international organizations are addressing the dual-use dilemma through preventive strategies. For instance, the World Health Organization (WHO) has published the “Global Guidance Framework for the Responsible Use of the Life Sciences”, emphasizing the importance of identifying and mitigating risks associated with the misuse of scientific knowledge (World Health Organization, 2022). Similarly, the Netherlands Biosecurity Office has developed the “Dual-use Quickscan tool” (www.dualusequickscan.com), designed to help researchers and institutions rapidly assess the dual-use potential of their projects. The tool consists of fifteen yes-or-no questions, grouped into three categories: (i) characteristics of the biological agent, (ii) knowledge and technology related to the agent, and (iii) potential consequences of misuse. Quickscan results provide a preliminary risk assessment that can serve as a foundation for discussions with biosafety and risk management advisors. Moreover, it can also be integrated into broader institutional biosafety and biosecurity programs, promoting continuous awareness and proactive monitoring of dual-use risks in academic and research settings (Vennis et al., 2021). On the other hand, the US has adopted a multi-layered strategy for managing dual-use research of concern through a combination of agency mandates, legislative actions, and institutional oversight. Notably, the “US Government Policy for Oversight of Dual Use Research of Concern and Pathogens with Enhanced Pandemic Potential” outlines a comprehensive framework that includes agent lists, institutional review, and federal oversight mechanisms (Government United States, 2024). Institutions such as the National Institute of Health (NIH) play an active role in implementing these policies. Additionally, the establishment of the National Security Commission on Emerging Biotechnology (NSCEB) reflects the country’s commitment to optimizing its dual-use research of concern governance. For instance, the NSCEB recently recommended that within the next year, the US Department of State should develop a new strategy for harmonizing multilateral export controls on conventional arms and dual-use technologies (National Security Commission on Emerging Biotechnology, 2025). Comparably, the European Union (EU) has pursued a precautionary approach, seeking to balance innovation with defense and responsible research. One key regulatory measure is the EU Regulation 2021/821 which establishes a unified control list of dual-use items across member states (European Parliament and Council, 2021). Additionally, the European Commission’s White Paper on “Options for enhancing support for research and development involving technologies with dual-use potential” emphasizes responsible innovations and cross-sector collaboration (Dual-use technologies, 2024). Another interesting example comes from China, which has focused on advancing innovation while strengthening biosecurity regulation. In 2020, China published its Biosecurity Law, establishing a legal foundation for biological research governance (Database, 2020). The Center for Strategic & International Studies (CSIS) has also documented the creation of China’s National Biotechnology Research and Development Safety Management Expert Committee, underscoring the country’s forward-looking stance on biotechnology oversight (Araz, 2020). Together, these examples reflect distinct but converging models of dual-use regulation and biosecurity preparedness, emphasizing institutional coordination, responsible innovation, and global governance.

### Role of scientific community and civil society in dual-use risk mitigation

In addition to governmental efforts, scientific communities and civil society organizations play a crucial role in enhancing dual-use awareness and fostering a culture of responsibility. Community-based initiatives help bridge educational, ethical, and institutional gaps, particularly in regions with underdeveloped regulatory infrastructure.

The previously mentioned iGEM competition serves as a compelling example of engagement. Since its outset, iGEM has emphasized that life sciences must be used responsibly. In 2021, iGEM further institutionalized this value by launching dedicated dual-use awareness workshop (IGEM, 2020). That same year, 1 out 15 teams that self-identified dual-use risks did so after attending one of these workshops, while three others had previously participated in similar workshops hosted by local synthetic biology associations. These educational efforts have even informed peer-reviewed publications documenting the international awareness gap around dual-use education (Vinke et al., 2022). Another notable example is the Youth for Biosecurity Fellowship, coordinated by the United Nations Office for Disarmament Affairs (UNODA) (UNODA, 2025). This initiative offers dual-use governance training to early career researchers from the Global South. Alumni of this initiative have gone on to contribute to national policy development, illustrating the long-term value of investing in biosecurity education as a component of scientific capacity-building. These examples demonstrate that building durable responses to dual-use risks requires multi-level engagement.

### Latin America challenges

Over time, the scientific community must integrate dual-use risk assessments as a core component of responsible research design. While international frameworks and tools are increasingly available, Latin America still faces significant challenges in adopting and implementing them effectively. This gap in implementation may be attributed to a combination of structural, institutional, and cultural factors. Although research specifically addressing these causes is limited, several plausible explanations can be identified:

• Perception of dual-use risks. At editorial level, some journal editors may not perceive dual-use risks as relevant or urgent. Others may lack the time or resource to develop appropriate policies, or may fear that such policies could restrict academic freedom or limit the open dissemination of knowledge. This perspective was further confirmed in a study published in 2014, which analyzed whether peer-reviewed life science journals from Brazil, Mexico, Argentina and Chile had any form of written policy addressing dual-use concerns. After reviewing the author and referee guidelines of 216 journals indexed in Latindex, the authors found that none of them included specific policies on dual-use concerns, even though many provided general ethical guidance on topics such as plagiarism, authorship, or the use of animals and human subjects (Valles and Bernacchi, 2014). This finding reveals a critical gap in editorial oversight regarding potentially sensitive scientific information in Latin America. More broadly, it reflects an institutional underestimation of dual-use risks, which likely hinders the development of governance and oversight mechanisms across the region.
• Nested regulatory layers. The complexity of overlapping legal frameworks is another key barrier. For instance, Argentina’s implementation of the Nagoya Protocol (Resolution 410/2019) required prior authorization to access genetic resources. However, because provinces were allowed to adapt the regulation locally, jurisdictional conflicts emerged, delaying access to scientific materials (Colmenarez et al., 2023). If dual-use regulations follow similar decentralized paths, it is reasonable to anticipate comparable legal fragmentation that could stall research at national and subnational levels.
• Overemphasis on containment, neglect of broader societal risks. Global biosafety governance has historically emphasized laboratory containment, particularly in high-containment labs, while overlooking broader issues related to knowledge misuse. This is in part because biosafety risks are tangible and easier to regulate, while misuse scenarios are more abstract and harder to legislate (Epstein, 2012; Novossiolova, 2017). Latin America now has an opportunity to develop forward-looking policies that balance immediate containment concerns with broader considerations of societal risk.
• Limited and fragmented funding. Progress in dual-use governance is often constrained by the lack of sustained financial support. In 2024, CRDF Global launched a small-grant competition to help members of the South American Research Security Consortium build research security and counter dual-use threats (CRDFGlobal, 2024). Also in 2024, the Organization of American States (OAS), the Inter-American Committee against Terrorism (CICTE), and the European Union provided €2.7 million to support biosafety and biosecurity training and regulatory harmonization in eight Latin American countries (OAS, 2025). While these efforts are promising, no long-term or region-wide funding programs currently exist to support the systematic development of dual-use-specific policies or regulatory frameworks.

Together, these barriers highlight the interconnected policy, perception, and capacity gaps that currently limit Latin America’s ability to address the dual-use dilemma. However, they also point to clear opportunities. By leveraging international tools, learning from global governance trends, and investing in regulatory harmonization, the region can build a more robust and responsible scientific culture. Future efforts should emphasize not only technical containment, but also the ethical and societal dimensions of research, ensuring that life sciences innovation continues to serve the public good.

## Conclusion

The dual-use dilemma highlights the need to balance scientific progress with the responsibility to prevent harm. While life sciences and emerging technologies like AI offer enormous benefits, they also carry risks of misuse, whether intentional or accidental. In Latin America, limited institutional awareness, regulatory gaps, and funding constraints make it urgent to strengthen governance, education, and oversight around dual-use research. Addressing these challenges requires coordinated action across governments, academia, and international partners to build a culture of responsibility that does not hinder innovation but ensures its safe and ethical application.

## Data Availability

The original contributions presented in the study are included in the article/supplementary material, further inquiries can be directed to the corresponding author.

## References

[B1] Araz S. (2020). China adopts biotechnology regulation, amid authoritarianism concerns. Cent. Strateg. Int. Stud. Available online at: https://www.csis.org/blogs/strategic-technologies-blog/china-adopts-biotechnology-regulation-amid-authoritarianism.

[B2] Bell J. A. Nuzzo J. B. (2021). Global health security index: advancing collective action and accountability amid global crisis. Available online at: www.GHSIndex.org (Accessed July 16, 2025).

[B3] Cello J. Paul A. V. Wimmer E. (2002). Chemical synthesis of poliovirus cDNA: generation of infectious virus in the absence of natural template. Science 297, 1016–1018. 10.1126/science.1072266 12114528

[B4] Colmenarez Y. C. Smith D. Walsh G. C. France A. Corniani N. Vásquez C. (2023). Regulatory frameworks for the access and use of genetic resources in Latin America. Neotrop. Entomol. 52, 333–344. 10.1007/s13744-022-01017-x 36729291 PMC11058676

[B5] CRDFGlobal (2024). Emerging research security leaders grant opportunities submission deadline. Available online at: https://www.crdfglobal.org/funding-opportunities/20424/ (Accessed July 16, 2025).

[B6] Database C. (2020). Biosecurity law of the PeopleʼS Republic of China.

[B7] Dual-use technologies (2024). Dual-use technologies. Available online at: https://research-and-innovation.ec.europa.eu/research-area/industrial-research-and-innovation/dual-use-technologies_en? (Accessed July 16, 2025).

[B8] Epstein G. L. (2012). Preventing biological weapon development through the governance of life science research. Biosecurity Bioterrorism 10, 17–37. 10.1089/bsp.2011.0091 22455676

[B9] European Parliament and Council (2021). Regulation (EU) 2021/821 of 20 May 2021 setting up a union regime for the control of exports, brokering, technical assistance, transit and transfer of dual-use items (recast). Off. J. Eur. Union 2021, 1–461.

[B10] Government United States (2024). United States government policy for oversight of dual use research of concern and pathogens with enhanced pandemic potential. Available online at: https://www.whitehouse.gov/wp-content/uploads/2024/05/USG-Policy-for-Oversight-of-DURC-and-PEPP.pdf (Accessed July 16, 2025).

[B11] IGEM (2020). Values and risks workshops. Available online at: https://responsibility.igem.org/opportunities/workshops (Accessed July 16, 2025).

[B12] Jackson R. J. Ramsay A. J. Christensen C. D. Beaton S. Hall D. F. Ramshaw I. A. (2001). Expression of mouse Interleukin-4 by a recombinant ectromelia virus suppresses cytolytic lymphocyte responses and overcomes genetic resistance to mousepox. J. Virol. 75, 1205–1210. 10.1128/jvi.75.3.1205-1210.2001 11152493 PMC114026

[B13] Miller S. Selgelid M. J. (2007). Ethical and philosophical consideration of the dual-use dilemma in the biological sciences. Sci. Eng. Ethics 13, 523–580. 10.1007/s11948-007-9043-4 18060518 PMC7089176

[B14] Millett P. Isaac C. R. Rais I. Rutten P. (2020). The synthetic-biology challenges for biosecurity: examples from iGEM. Nonproliferation Rev. 27, 443–458. 10.1080/10736700.2020.1866884

[B15] National Security Commission on Emerging Biotechnology (2025). Charting the future of biotechnology.

[B16] Novossiolova T. (2017). “Twenty-first century governance challenges in the life sciences,” in Governance of biotechnology in post-soviet Russia, 113–165. 10.1007/978-3-319-51004-0_4

[B17] OAS (2025). Strengthening biosafety and biosecurity in Latin America. A Summ. main results EU-funded OAS/CICTE Proj. Available online at: https://www.oas.org/es/sms/cicte/docs/StrengtheningBiosafetyandBiosecurityinLatinAmerica_Asummaryofthemainresults.pdf (Accessed July 16, 2025).

[B18] Razavi A. Erondu N. Okereke E. (2020). The global health security index: what value does it add. BMJ Glob. Heal. 5, e002477–3. 10.1136/bmjgh-2020-002477 PMC7213809 32349994

[B19] Sandbrink J. B. Alley E. C. Watson M. C. Koblentz G. D. Esvelt K. M. (2023). Insidious insights: implications of viral vector engineering for pathogen enhancement. Gene Ther. 30, 407–410. 10.1038/s41434-021-00312-3 35264741 PMC10191845

[B20] Selgelid M. J. (2009). Governance of dual-use research: an ethical dilemma. Bull. World Health Organ. 87, 720–723. 10.2471/BLT.08.051383 19784453 PMC2739909

[B21] UNODA (2025). Apply for the 2025 youth for biosecurity fellowship. Available online at: https://disarmament.unoda.org/youth4biosec-fellowship-2025/ (Accessed July 16, 2025).

[B22] Urbina F. Lentzos F. Invernizzi C. Ekins S. (2022). Dual use of artificial-intelligence-powered drug discovery. Nat. Mach. Intell. 4, 189–191. 10.1038/s42256022004659 36211133 PMC9544280

[B23] Valles E. G. Bernacchi A. S. (2014). Do latin American scientific journals follow dual-use review policies? Biosecurity Bioterrorism 12, 94–105. 10.1089/bsp.2013.0088 24693885 PMC3993063

[B24] Vennis I. M. Schaap M. M. Hogervorst P. A. M. de Bruin A. Schulpen S. Boot M. A. (2021). Dual-use quickscan: a web-based tool to assess the dual-use potential of life science research. Front. Bioeng. Biotechnol. 9, 797076–797079. 10.3389/fbioe.2021.797076 34957083 PMC8696162

[B25] Vinke S. Rais I. Millett P. (2022). The dual-use education gap: awareness and education of life science researchers on nonpathogen-related dual-use research. Heal. Secur. 20, 35–42. 10.1089/hs.2021.0177 35175856

[B26] World Health Organization (2022). Global guidance framework for the responsible use of the life sciences. Available online at: https://www.who.int/publications/i/item/9789240056107 (Accessed July 16, 2025).

